# Tumor Location and Preoperative Biliary Stenting Shape Gut Microbiome Diversity in Pancreatic Cancer

**DOI:** 10.3390/cancers18162617

**Published:** 2026-08-14

**Authors:** Marionna Cathomas, Franco Fortunato, Eli Zamir, Marisa Isabell Keller, Toohina Gobin, Laila Jötten, Elias Gauer, Max Heckler, Bo Kong, Rogier Aäron Gaiser, Ingmar F. Rompen, Jonathan M. Harnoss, Sabine Schmidt, Michael Kuhn, Eran Elinav, Peer Bork, Christoph W. Michalski, Thomas Hank

**Affiliations:** 1 Department of General, Visceral and Transplantation Surgery, Heidelberg University Hospital, 69120 Heidelberg, Germany; franco.fortunato@med.uni-heidelberg.de (F.F.); bo.kong@med.uni-heidelberg.de (B.K.);; 2 Division of Microbiome and Cancer, German Cancer Research Center (DKFZ), 69120 Heidelberg, Germany; 3 Molecular Systems Biology Unit, European Molecular Biology Laboratory (EMBL), 69117 Heidelberg, Germany; 4 Department of Systems Immunology, Weizmann Institute of Science, Rehovot 76100, Israel

**Keywords:** pancreatic ductal adenocarcinoma, preoperative biliary stenting, intestine microbiome

## Abstract

**Background**: Recent evidence suggests that gut microbiome plays a role in the development of pancreatic ductal adenocarcinoma (PDAC) and influences treatment response. However, the association of tumor location and preoperative biliary stenting (PBS) with gut microbial composition and diversity remains poorly understood. **Methods**: Preoperative stool specimens were prospectively collected from patients with PDAC undergoing surgery between March 2020 and July 2021 at the Department of Surgery, Heidelberg University Hospital, Germany. Whole-genome shotgun metagenomic sequencing was performed. Microbial diversity was assessed using the Shannon index and Bray–Curtis dissimilarity with principal coordinates analysis. **Results**: A total of 63 preoperative stool samples were analyzed from 40 patients with pancreatic head (63.5%) and 23 with body/tail tumors (36.5%). Baseline characteristics were comparable between groups. Microbial community composition differed significantly between tumor locations (Bray–Curtis, *p* = 0.005), with enrichment of *Ruminococcus bromii* in body/tail tumors. Among patients with pancreatic head tumors, PBS was associated with reduced alpha diversity (Shannon index, *p* = 0.04) and depletion of taxa including *Eubacteriales* and *Clostridiales taxa*, and members of the genera *Raoultella* and *Prevotella*. PBS was associated with a higher rate of major complications > 3a according to the Clavien–Dindo classification (28.6% vs. 3.8%; *p* = 0.04). **Conclusions**: PBS was associated with reduced microbial diversity and distinct taxonomic alterations of the gut microbiome. These findings suggest that biliary stenting is associated with microbiome alterations that may be relevant for perioperative risk stratification and warrant further investigation.

## Simple Summary

In general, the gut microbiome—the composition of bacteria and other microorganisms in the gut—is known to influence the postoperative and oncological outcomes of malignant tumors. However, less is known about its specific role in pancreatic cancer. The present study examined changes in the microbiome according to tumor location within the pancreas (pancreatic head vs. pancreatic body/tail) and demonstrated significant differences in microbial community composition. Furthermore, a subgroup analysis of patients with pancreatic head cancer investigated the association between preoperative biliary stenting and gut microbiome characteristics. The results showed reduced microbial diversity in patients with preoperative stent placement, which was associated with worse postoperative outcomes. These findings suggest that clinical factors such as tumor location and biliary stenting are associated with distinct microbiome patterns in PDAC and warrant further investigation regarding their potential relevance for perioperative risk stratification and patient outcomes.

## 1. Introduction

The gut microbiome has emerged as an important modulator of host physiology and immune responses, with increasing evidence linking microbial alterations to tumor progression and adverse postoperative outcomes [1,2,3]. Disruptions in microbial homeostasis can impair intestinal barrier function, promote bacterial translocation, and alter host–microbiome interactions, thereby contributing to systemic inflammation and disease progression [4]. Current evidence suggests that gut microbiome composition influences not only cancer development but also treatment response, postoperative recovery, and long-term oncological outcomes. In particular, reduced microbial diversity and shifts in microbial community composition have been associated with unfavorable clinical outcomes in several gastrointestinal malignancies. While these mechanisms have been extensively studied in colorectal surgery, their role in pancreatic ductal adenocarcinoma (PDAC) remains incompletely understood [5,6].

In PDAC, tumor location is associated with distinct clinical and biological contexts [7]. Tumors of the pancreatic head frequently present with obstructive jaundice. Obstructive jaundice may result in impaired liver function and often necessitates preoperative biliary stenting (PBS) [8]. Several studies have demonstrated an association between PBS and adverse postoperative outcomes; however, the underlying mechanisms remain poorly understood. In addition, previous investigations have shown that biliary stenting alters the microbial composition of bile and may influence intestinal microbial communities. These observations suggest that PBS may represent an important iatrogenic factor shaping the gut microbiome in patients with PDAC [9].

In contrast to PDAC located in the pancreatic head, tumors of the pancreatic body and tail typically develop without biliary obstruction, representing a different physiological environment.

Despite advances in surgical techniques and perioperative management, PDAC remains one of the most lethal malignancies worldwide. Furthermore, postoperative morbidity remains substantial. Given the high postoperative morbidity and the dismal prognosis associated with PDAC, a better understanding of microbiome-associated mechanisms may identify modifiable targets to improve perioperative and, therefore, long-term outcomes [10]. However, it remains unclear whether differences related to tumor location and PBS are primarily reflected at the taxonomic level, in microbial diversity, or in microbial functional capacity. This study aimed to characterize taxonomic differences in the gut microbiome of PDAC patients according to tumor location and to determine microbiome alterations associated with PBS.

## 2. Materials and Methods

### 2.1. Study Design and Population

This observational cohort study included 63 patients with PDAC located in the pancreatic head or body/tail, who underwent surgery between March 2020 and July 2021 at the Department of Surgery, Heidelberg University Hospital, Germany. Demographic and clinical data were retrieved from a prospectively maintained institutional database.

The study was approved by the Ethics Committee of Heidelberg University (S-141/2019). All patients provided informed consent.

### 2.2. Follow-Up and Clinical Variables

The primary endpoint was the assessment of differences in gut microbiota diversity between patients with PDAC located in the pancreatic head and those with body/tail tumors. A predefined subgroup analysis focused on patients with PDAC of the pancreatic head according to the presence of PBS, including an evaluation of microbial functional capacity with respect to secondary bile acid metabolism.

Patients received a single dose of antibiotics prior to PBS. In addition, patients undergoing PBS received an additional course of antibiotics on the day before surgery. By that time, however, the stool samples had already been collected.

Postoperative complications were defined as any medical or surgical condition deviating from the normal postoperative course and were graded according to the Clavien–Dindo classification [11]. Complications graded ≥ 3a were considered major complications. All complications occurring within 30 days after hospital discharge, including reoperations and readmissions, were recorded.

### 2.3. Collection and Microbiome Sequencing

Patients were recruited during outpatient consultation. After informed consent, stool samples were self-collected at home at least three days, but no more than 6 days, prior to surgery using a standardized collection kit and stored in stored in Zymo DNA/RNA Shield™ solution. With the Zymo DNA/RNA Shield™ solution, the samples could be stored at room temperature until processing. At the time of hospital admission, participants brought the previously collected samples to the study site for performing metagenomic sequencing. DNA extraction was performed using the Qiagen AllPrep PowerFecal DNA/RNA Kit (Qiagen, Hilden, Germany) with an additional washing step with phosphate-buffered saline at the beginning to remove the Zymo solution. To generate sequencing libraries, the NEBNext Ultra II DNA library prep kit (New England Biolabs, Ipswich, MA, USA) was used together with dual index multiplex oligos. A total of 2 × 150 bp paired-end sequencing reads were acquired on an Illumina HiSeq 4000 platform (Illumina, San Diego, CA, USA).

### 2.4. Sequencing Data Analysis

Raw sequencing reads were quality-filtered using fastp (version 0.23.4) to remove low-quality reads and reads shorter than 50 bp. Human contaminant reads were removed using Bowtie2 (version 2.3.5.1) with the hg38 reference genome and samtools (version 1.5). Remaining reads were rarefied to 25 million reads per sample using seqtk (version 1.4-r122). Taxonomic profiling was performed using Kraken (version 2.1.3), followed by species abundance re-estimation with Bracken (version 2.9), based on a Kraken database including human, mouse, and fungal GTDB references.

### 2.5. Microbiome Data Analysis

Taxa with relative abundances below predefined thresholds were filtered as specified for each analysis, and remaining values below the threshold were set to the threshold value. Alpha diversity was calculated using the Shannon index as implemented in the shannon function of the skbio.diversity.alpha Python package (0.6.0). Beta diversity was assessed after abundance filtering (threshold 0.001) using Bray–Curtis dissimilarity and visualized by principal coordinate analysis (PCoA) with the skbio.stats.distance and skbio.stats.ordination modules.

Differential abundance analysis was performed after abundance filtering (threshold 0.01) using the Mann–Whitney U test. A total of 246 species met the abundance-filtering criteria and were included in the differential abundance analysis. Species with a *p*-value < 0.05 and a fold change >1.3 were considered significant. The differential abundance analysis was applied on data which was normalized to a sum of 1 per sample, without further subjecting it to centered log-ratio (CLR) transformation. The testing was based on a non-parametric test (Mann–Whitney U rank test). For subgroup analyses, microbial functional potential was assessed using Kyoto Encyclopedia of Genes and Genomes (KEGG) pathway analysis. Secondary bile acid metabolism was evaluated using KEGG pathway ko00121, focusing on K01442 (bile salt hydrolase), K07007 (baiN), and K15969 (baiB).

### 2.6. Statistical Analysis

Statistical analyses were performed using SPSS Statistics (version 30). Continuous variables were analyzed using Student’s *t*-test or Mann–Whitney U test, as appropriate, and are presented as mean or median values. Categorical variables were compared using the chi-square test or Fisher’s exact test, as indicated. To identify factors independently associated with reduced microbial diversity, a multivariable binary logistic regression model was constructed with low alpha diversity as the dependent variable. Covariates included BMI, diabetes mellitus, bilirubin level, CA19-9 concentration, intraoperative smear status, and preoperative biliary stenting. Results are presented as odds ratios (ORs) with 95% confidence intervals (CIs). A two-sided *p* value < 0.05 was considered statistically significant.

Analysis of variance (ANOVA) was applied to assess differences in Shannon diversity. Differences in alpha diversity were additionally evaluated using the Mann–Whitney U test as implemented in the scipy.stats module. Differences in beta diversity were assessed using permutational multivariate analysis of variance (PERMANOVA) implemented in skbio.stats.distance. Functional analysis was performed on KEGG-PATHWAY and keg-orthologous genes (KO) (prevalence ≥ 0.05, abundance ≥ 1 × 10^−5^) using Wilcoxon rank sum test with subsequent false discovery rate (fdr) correction for multiple testing.

## 3. Results

### 3.1. Patient Cohort and Characteristics

A total of 63 patients with PDAC were included, comprising tumors located in the pancreatic head (n = 40) and body/tail (n = 23). Baseline demographic and tumor-related characteristics were comparable between groups (Table 1 and Table 2). Patients with pancreatic head tumors underwent more complex surgical procedures and had longer operative times (*p* = 0.024). Major postoperative complications > 3a according to the Clavien–Dindo classification occurred more frequently in patients with pancreatic head tumors (17.5% vs. 0%; *p* = 0.031), with a corresponding increase in 90-day mortality (7.5% vs. 0; *p* = 0.546).

### 3.2. Microbiome Differences by Tumor Location

**Figure 1:**
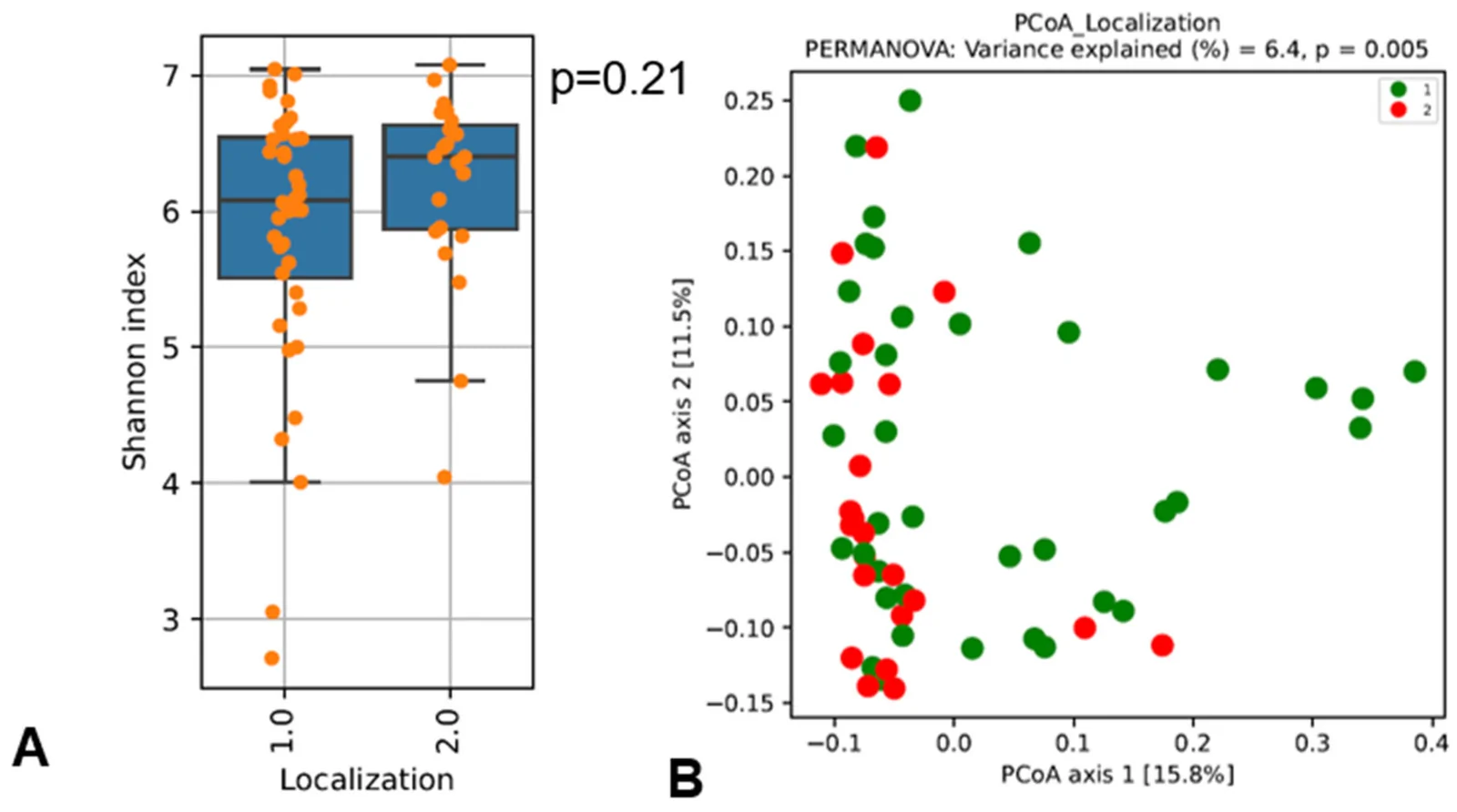
Alpha diversity (**A**) measured by Shannon index and beta diversity (**B**) analyzed by principal coordinate analysis (PCoA) based on species-level Bray–Curtis dissimilarity. PDAC head (1.0) and PDAC body/tail (2.0); PERMANOVA, permutational multivariate analysis of variance.

**Figure 2:**
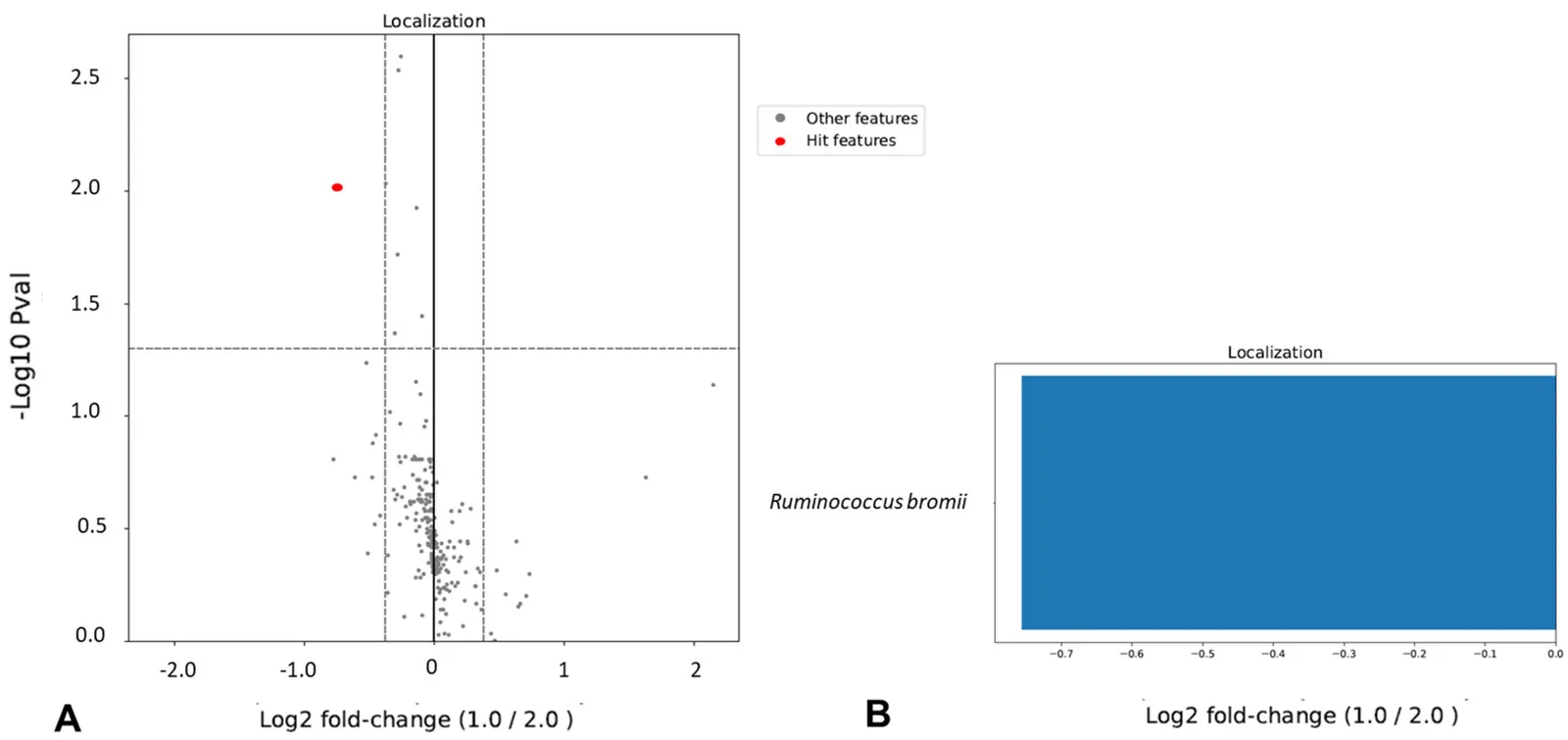
(**A**) Relative abundance in PDAC head (1.0) and PDAC body/tail (2.0). (**B**) Taxa with significantly higher abundance in patients with PDAC body/tail.

Microbial alpha diversity (Shannon index) of stool samples did not differ between pancreatic tumor locations (*p* = 0.21; Figure 1A). In contrast, microbial community composition differed significantly between groups, as assessed by Bray–Curtis dissimilarity (*p* = 0.005; Figure 1B). Exploratory differential abundance analysis identified an enrichment of *Ruminococcus bromii* in patients with pancreatic body/tail tumors (Figure 2).

### 3.3. Clinical Correlations of Tumor Locations and PBS

Among patients with PDAC located in the head, 14 (35.0%) underwent PBS. Baseline characteristics and surgical variables were comparable between patients with and without PBS (Supplementary Tables S1 and S2). Further, all known influencing factors as metformin or proton pump inhibitor (PPI) were comparable among the two groups. The median interval between PBS and surgery was 29 days (IQR: 18.0–66.5). Patients with PBS experienced a higher rate of major postoperative complications compared with those without PBS (28.6% vs. 3.8%; *p* = 0.04). Intraoperative bile cultures, available in 67.5% of patients, were more frequently positive in the PBS group (85.7% vs. 42.3%; *p* < 0.001), with predominantly polymicrobial growth (Table 3).

### 3.4. Microbiome Alterations Associated with PBS

**Figure 3:**
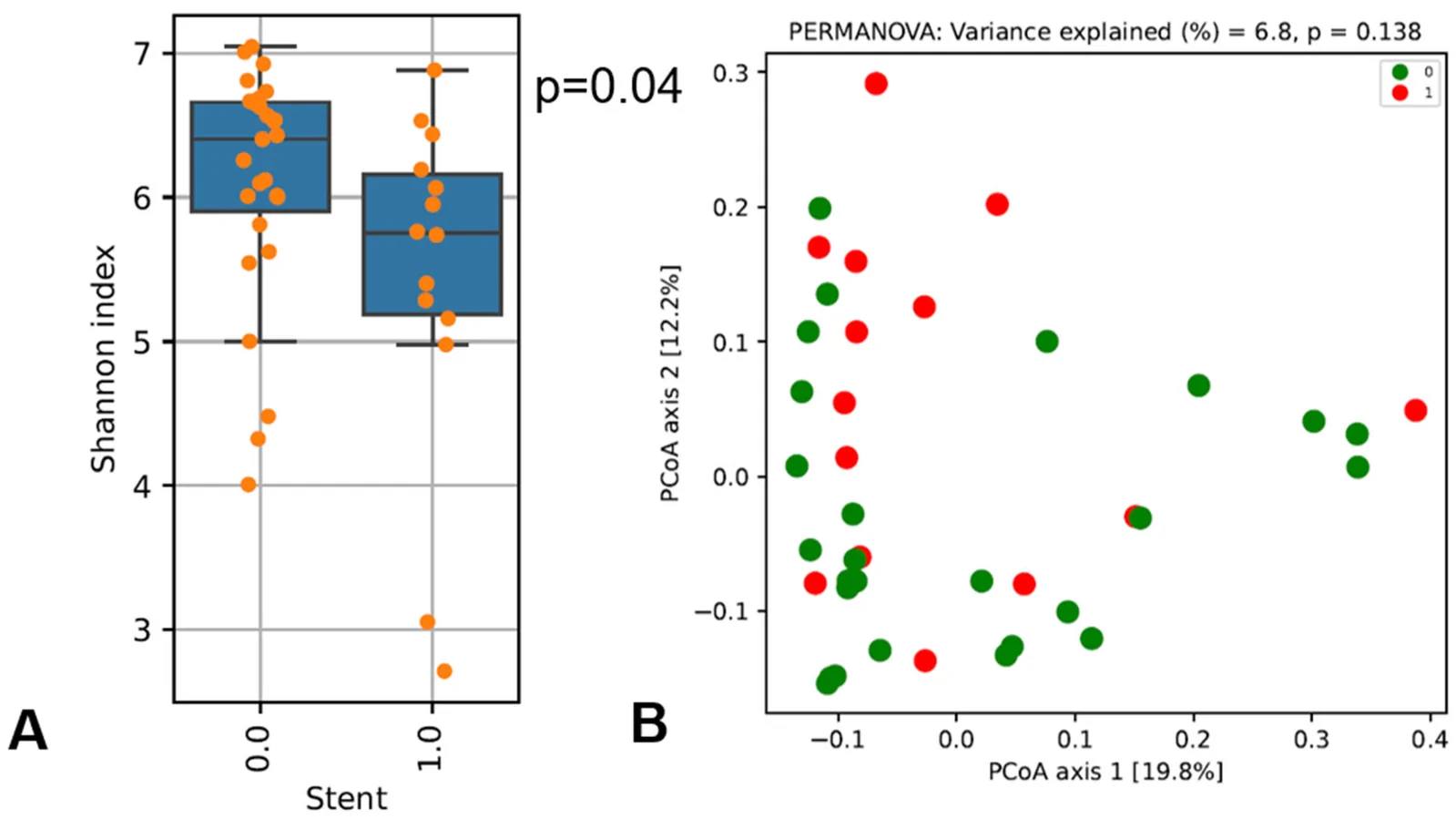
Alpha diversity (**A**) measured by Shannon index and beta diversity (**B**) analyzed by principal coordinate analysis (PCoA) based on species-level Bray–Curtis dissimilarity. PDAC head without (0.0) and with (1.0) preoperative biliary stent; PERMANOVA, permutational multivariate analysis of variance.

**Figure 4:**
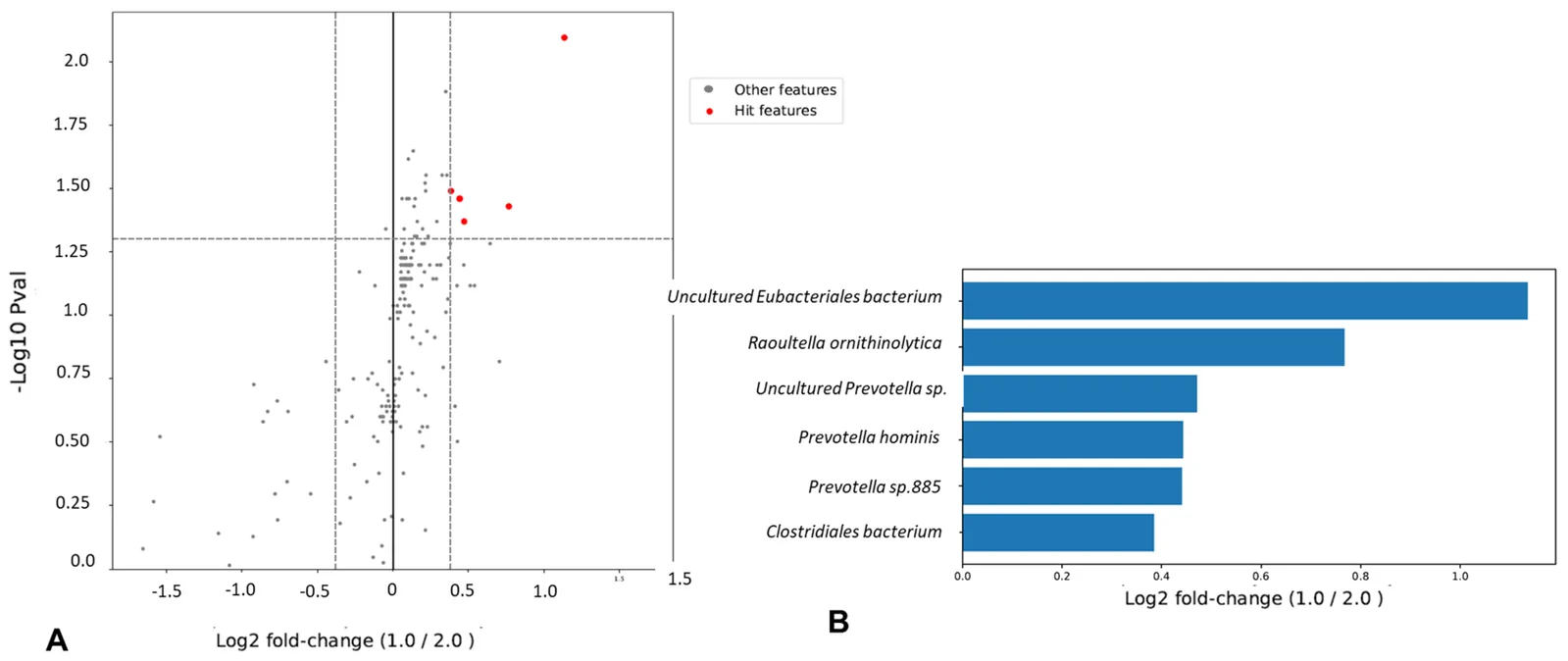
(**A**) Relative abundance in patients without (0.0) and with (1.0) preoperative biliary stent. (**B**) Taxa with significance higher abundance in patients without preoperative biliary stent.

PBS was associated with reduced alpha diversity (*p* = 0.04; Figure 3A), whereas no significant difference in beta diversity was observed (*p* = 0.138; Figure 3B). Taxonomic analysis demonstrated a relative depletion of several bacterial taxa, including *uncultured Eubacteriales bacterium*, *Clostridiales bacterium*, and *Prevotella* species, in patients with PBS (Figure 4). In multivariable logistic regression analysis, PBS remained the only independent factor associated with low alpha diversity (OR 8.9, 95% CI 1.1–74.0, *p* = 0.04; Supplementary Table S3).

### 3.5. Functional Microbiome Profiling

**Figure 5:**
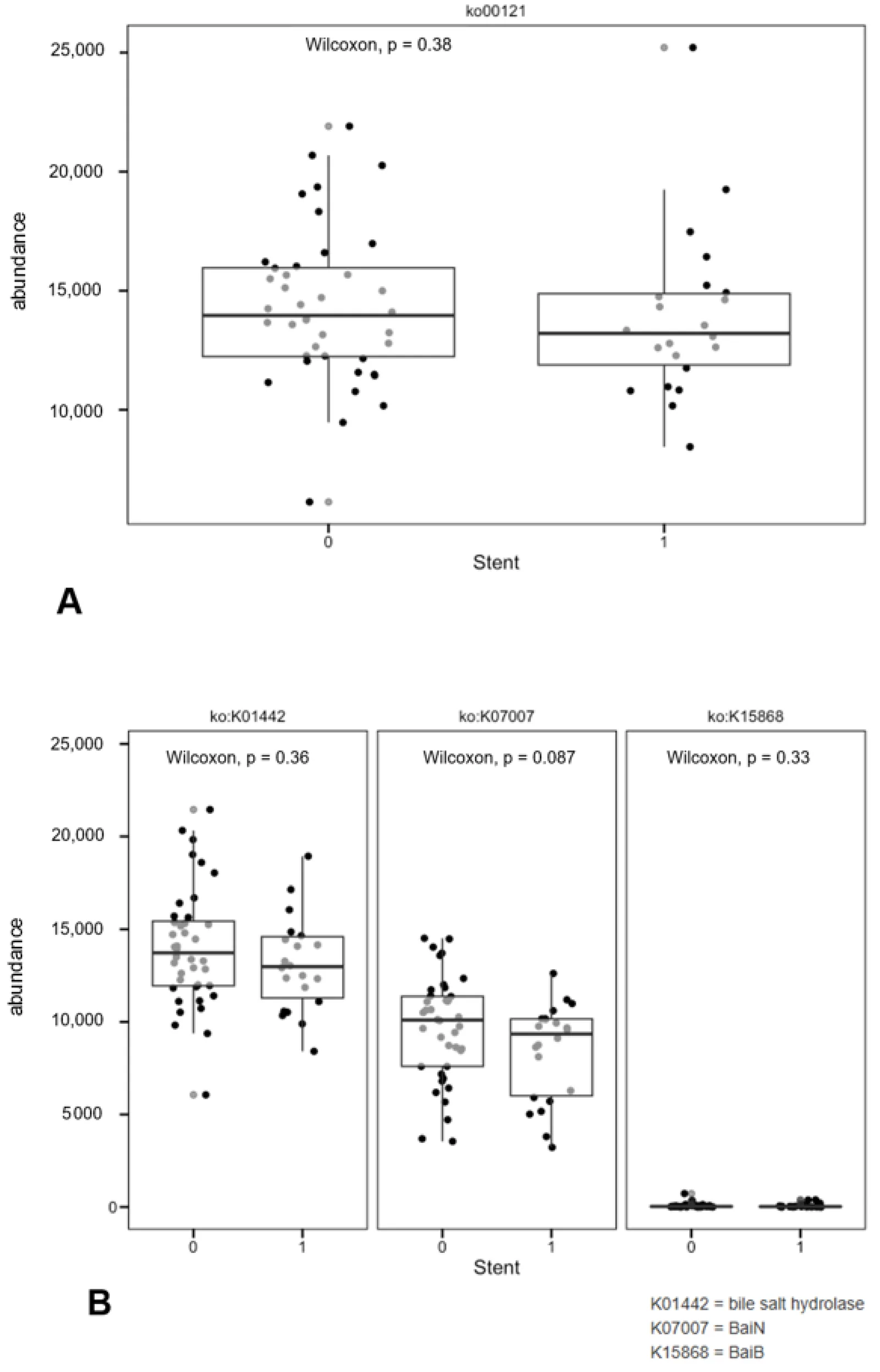
Secondary bile acid metabolism using Kyoto Encyclopedia of Genes and Genomes (KEGG) pathway (**A**). Functional activity was measured by K01442 (bile salt hydrolase), K07007 (baiN) and K15969 (baiB) (**B**). Assessment was performed in patients with and without preoperative biliary stent (PBS).

To further explore the biological relevance of these microbial alterations, functional pathway analysis was performed on 4443 KEGG orthologous genes. We found no significantly changing genes after correcting for multiple testing. Given the potential influence of PBS on bile acid composition within the intestinal lumen, we specifically examined bile acid-related KOs and functional pathway abundances. Functional pathway analysis revealed no change in the secondary microbial bile acid metabolism pathway (map00121) in patients with PBS (Figure 5A). Likewise, individual KO within the pathways, the baiN-associated gene (K07007), the bile salt hydrolase (K01442) or the baiB gene (K15969) showed no difference (Figure 5B). These findings indicate that no significant differences in secondary microbial bile acid metabolism were detected between patients with and without PBS.

## 4. Discussion

In this study, we demonstrate that tumor location and PBS are associated with distinct alterations of the preoperative gut microbiome in PDAC. While tumors located in the pancreatic body and tail were characterized by differences in microbial community composition, PBS in patients with pancreatic head tumors was associated with reduced within-patient microbial diversity, while no significant alterations in bile acid-related microbial functions were observed after correction for multiple testing. These findings suggest that tumor-related and iatrogenic factors are linked to distinct microbiome alterations with potential clinical relevance.

Patients with PDAC located in the pancreatic body and tail showed significantly different beta diversity compared with those with tumors in the pancreatic head, while alpha diversity remained comparable. This indicates that tumor location primarily affects microbial community composition rather than overall microbial richness. The observed enrichment of *Ruminococcus bromii* in body/tail tumors is noteworthy, as this species plays a central role in the degradation of resistant starch and supports the production of short-chain fatty acids such as butyrate [12]. Through these mechanisms, *Ruminococcus bromii* has been linked to microbial stability, maintenance of epithelial barrier integrity, and modulation of immune responses. Accordingly, the presence of such taxa may reflect a microbial profile more consistent with gut homeostasis [13].

These location-dependent microbial differences are biologically plausible. Pancreatic head tumors typically arise in a biliary-influenced environment and are frequently associated with cholestasis and endoscopic interventions, whereas body and tail tumors develop largely independent of direct biliary exposure. These findings suggest a more gut-derived microbial profile in body/tail tumors, while head tumors appear to be more strongly influenced by biliary conditions [8,14,15,16,17]. While surgical complexity likely explains the higher complication rates observed in patients with pancreatic head tumors, microbiome-associated factors may additionally contribute to postoperative vulnerability [18].

Notably, beta diversity remained comparable, suggesting a global depletion of microbial diversity rather than a distinct microbial community shift. This pattern may be consistent with a microbiome profile suggestive of dysbiosis, potentially associated with retrograde bacterial translocation, biofilm formation or prolonged exposure of the biliary system to intestinal microbes [19]. Because patients undergoing PBS also received a single antibiotic dose at the time of stent placement, we cannot completely exclude an influence of antibiotic exposure on the observed microbiome changes. The median interval between PBS and surgery was 29 days, although substantial variability existed between patients. Future studies should evaluate the potential impact of the interval between biliary intervention and microbiome sampling in greater detail.

Importantly, no significant differences in secondary bile acid metabolism pathways or bile acid-related gene abundances were detected between patients with and without PBS after correction for multiple testing [17]. Secondary bile acids play a key role in maintaining intestinal homeostasis and regulating inflammatory responses [20,21]. The absence of detectable alterations in bile acid-related microbial functions despite reduced microbial diversity may suggest a degree of functional resilience within the gut microbiome; however, this interpretation remains speculative and requires validation in larger cohorts and through direct metabolomic analyses [22,23]. Furthermore, given the relatively small sample size, particularly within the PBS subgroup, subtle functional differences cannot be excluded. Together, these findings suggest that global microbiome alterations may be more informative than the presence of individual microbial taxa or pathogens alone.

These findings are in line with previously published data demonstrating that PBS is associated with altered biliary and intestinal microbiota and higher rates of major complications [24]. While causality cannot be established, the association between reduced microbial diversity and adverse clinical outcomes suggests that microbiome changes may reflect a vulnerable perioperative state. Importantly, no significant alterations in bile acid-related microbial functions were observed, indicating that the functional consequences of reduced microbial diversity remain uncertain [25,26].

From a clinical perspective, both tumor location and PBS are identifiable preoperatively, but only PBS represents a potentially modifiable factor [27]. Beyond patient selection for biliary drainage, the growing interest in microbiome-targeted interventions may provide additional opportunities for perioperative optimization in the future. Approaches such as dietary modulation, probiotics, prebiotics, or fecal microbiota-based strategies have shown promise in other clinical settings and may eventually contribute to reducing postoperative morbidity. Current guidelines already recommend restrictive use of preoperative biliary drainage; our findings provide additional biological context supporting this approach. In the future, microbiome-based risk stratification and targeted perioperative interventions may represent promising strategies, although prospective studies are required [8,28].

This study has several limitations. The cohort size is limited, particularly for subgroup analyses, and the observational design precludes causal inference. Stool-based microbiome profiling does not capture site-specific microbial dynamics. Dietary intake and other environmental factors influencing the microbiome could not be fully controlled. In addition, functional predictions based on metagenomic data require validation by metabolomic analyses. Moreover, the absence of non-PDAC controls limits conclusions regarding disease-specific microbial signatures. Accordingly, our findings should be interpreted as differences within PDAC according to tumor location and PBS. Another limitation of this study is that differential abundance analyses were performed on relative abundance data without applying compositional data transformations such as centered log-ratio (CLR). Although this approach is commonly used in exploratory microbiome research, it may be susceptible to compositional effects. Furthermore, taxonomic differential abundance analyses were not adjusted for multiple testing and should therefore be regarded as exploratory. Finally, the absence of significant functional differences after correction for multiple testing may in part reflect limited statistical power related to the relatively small cohort size, particularly in the PBS subgroup.

## 5. Conclusions

In conclusion, tumor location and PBS are associated with distinct alterations of the gut microbiome in PDAC. While tumor location primarily influences microbial composition, biliary stenting is linked to reduced microbial diversity. These findings support a potential interaction between clinical factors and the gut microbiome and suggest that microbiome alterations associated with biliary intervention may represent a modifiable biological pathway linking clinical practice to postoperative risk. Although the functional consequences of these microbial alterations remain incompletely understood, the observed associations highlight the relevance of the gut microbiome as a potential contributor to perioperative outcomes in pancreatic cancer. Future prospective studies with larger cohorts and longitudinal sampling strategies are warranted to validate these findings and to explore whether microbiome-targeted interventions may improve patient outcomes. Nevertheless, to our knowledge, this study represents one of the first investigations specifically addressing the relationship between tumor location, preoperative biliary stenting, and gut microbiome alterations in PDAC, thereby providing a foundation for future research in this emerging field.

## Figures and Tables

**Table 1:** Patient characteristics of the cohort.

	All Patients (n = 63)	PDAC Head (n = 40)	PDAC Body/Tail (n = 23)	*p*-Value
Age (median; IQR)	64 (43–80)	64 (48–80)	66 (43–77)	0.721
Gender (male)	43 (68.3%)	28 (70.0%)	15 (65.2%)	0.695
Weight (median; IQR)	78 (48–105)	78.1 (48–105)	77.3 (55–103.3)	0.618
BMI (median; IQR)	25.3 (17.6–37.9)	25.4 (17.6–32.5)	25.1 (18.8–37.9)	0.618
ASA				
2	38 (60.3%)	23 (57.5%)	15 (65.2%)	0.547
3	25 (39.7%)	17 (42.5%)	8 (34.8%)	
Diabetes				
No	44 (69.8%)	27 (67.5%)	17 (73.9%)	0.593
Yes	13 (31.7%)	13 (32.5%)	19 (26.1%)	
Metformin				0.498
No	52 (82.5%)	34 (85.0%)	18 (78.3%)	
Yes	11 (17.5%)	6 (15.0%)	5 (21.7%)	
PPI				0.270
No	44 (69.8%)	26 (65.0%)	18 (78.3%)	
Yes	19 (30.2%)	14 (35.0%)	5 (21.7%)	

BMI, body mass index in kg/m^2^; ASA, grading in American Society of Anesthesiologists; PPI, proton pump inhibitor.

**Table 2:** Surgical and clinical outcomes.

	All Patients (n = 63)	PDAC Head (n = 40)	PDAC Body/Tail (n = 23)	*p*-Value
CRP (preoperative; median; IQR)	2.4 (4–57.5)	2.4 (1.0–54.0)	2.8 (4–57.5)	0.721
Bilirubin (preoperative; median; IQR)	0.6 (0.1–13.9)	0.7 (0.3–13.9)	0.5 (0.1–1.9)	0.063
CA 19-9 (preoperative; median; IQR)	54.9 (0.09–50,511)	59.9 (90–50,511)	54.9 (0.09–1313.60)	0.868
Tumor stage				0.541
I	19 (30.2%)	12 (30.0%)	7 (30.4%)	
II	13 (20.6%)	10 (25.0%)	3 (13.0%)	
III	17 (27.0%)	11 (27.5%)	6 (26.1%)	
IV	14 (22.2%)	7 (17.5%)	7 (30.4%)	
Neoadjuvant chemotherapy				0.991
No	52 (82.5%)	33 (82.5%)	19 (82.6%)	
Yes	11 (17.5%)	7 (17.5%)	4 (17.4%)	
Surgery				<0.001
Partial pancreatoduodenectomy	28 (44.4%)	28 (70.0%)	0	
Distal pancreatectomy	13 (20.6%)	0	13 (56.5%)	
Total pancreatectomy	12 (19.0%)	6 (15.0%)	6 (26.1%)	
Exploration	10 (15.9%)	6 (15.0%)	4 (17.4%)	
Duration of the surgical procedure (median, in min; IQR)	330 (24–662)	373 (36–662)	279.5 (24–487)	0.024
Blood loss (median, in ml; IQR)	800 (0–6000)	800 (0–6000)	750 (0–4500)	0.828
Complications *				0.041
>3a	7 (11.1%)	7 (17.5%)	0	
90-day mortality	3 (4.8%)	3 (7.5%)	0	0.546

CA 19-9, carbohydrate antigen 19-9; CRP, C-reactive protein * According to Clavien–Dindo classification within 30 days after discharge.

**Table 3:** Microbiological analysis of positive bile culture in patients with and without preoperative biliary stent (PBS).

	Overall	With PBS	Without PBS
Positive bile culture	23 (57.5%)	12 (85.7%)	11 (42.3%)
*Escherichia coli*	6 (26.1%)	3 (25.0%)	3 (27.3%)
*Enterococcus faecalis*	10 (43.5%)	7 (58.3%)	3 (27.3%)
*Enterococcus faecium*	4 (17.4%)	4 (33.3%)	0
*Klebsiella pneumoniae*	1 (4.3%)	1 (8.3%)	0
*Other Klebsiella species*	2 (8.7%)	2 (16.7%)	0
*Streptococcus species*	3 (13.0%)	3 (25.0%)	0
*Candida species*	3 (13.0%)	3 (25.0%)	0
Others	5 (21.7%)	5 (41.7%)	0

## Data Availability

The original data presented in the study are openly available.

## Supplementary Materials

The following supporting information can be downloaded at https://www.mdpi.com/article/10.3390/cancers18162617/s1, Table S1: Patient characteristics of the cohort with pancreatic head cancer. Table S2: Surgical and clinical outcomes with pancreatic head cancer. Table S3: Multivariable analysis to analyse independent factor for low alpha diversity.

## Abbreviations

The following abbreviations are used in this manuscript:

ANOVA	Analysis of variance
CLR	Centered log-ratio
DNA	Deoxyribonucleic acid
KEGG	Kyoto Encyclopedia of Genes and Genomes
GTDB	Genome Taxonomy Database
FDR	False discovery rate
PBS	Preoperative biliary stenting
PDAC	Pancreatic ductal adenocarcinoma
PERMANOVA	Permutational multivariate analysis of variance

## References

[1] Liang M., Liu Z., Zhang R., Yang J.H., Wang X.W., Zhang N. (2025). Changes in intestinal microbiota in patients with pancreatic cancer: A systematic review and meta-analysis. Front. Microbiol..

[2] Schmitt F.C.F., Brenner T., Uhle F., Loesch S., Hackert T., Ulrich A., Hofer S., Dalpke A.H., Weigand M.A., Boutin S. (2019). Gut microbiome patterns correlate with higher postoperative complication rates after pancreatic surgery. BMC Microbiol..

[3] Nichols L., El-Kholy O., Elsayed A.A.R., Basson M.D. (2025). The bidirectional interplay between gut dysbiosis and surgical complications: A systematic review. Am. J. Surg..

[4] Zheng Z., Hu Y., Tang J., Xu W., Zhu W., Zhang W. (2023). The implication of gut microbiota in recovery from gastrointestinal surgery. Front. Cell. Infect. Microbiol..

[5] Helliwell J.A., Chilton C.H., Young C., Clark E.V., Wilkinson L., Balaguer A.F., Bottomley D., Croft J., Corrigan N., Kirby A. (2025). Perioperative changes in the microbiome during rectal cancer surgery: Exploratory analysis of the National Institute for Health and Care Research (NIHR) IntAct trial. Br. J. Surg..

[6] Kartal E., Schmidt T.S.B., Molina-Montes E., Rodríguez-Perales S., Wirbel J., Maistrenko O.M., Akanni W., Alhamwe B.A., Alves R.J., Carrato A. (2022). A faecal microbiota signature with high specificity for pancreatic cancer. Gut.

[7] Park W., Chawla A., O’Reilly E.M. (2021). Pancreatic Cancer: A Review. JAMA.

[8] Klotz R., Hank T., Berente M.P., Joos M., Hinz U., Pianka F., Kinny-Köster B., Al-Saeedi M., Strobel O., Hackert T. (2026). Preoperative Surgical or Endoscopic Bile Duct Drainage in Pancreatic Cancer. Ann. Surg..

[9] van der Gaag N.A., Rauws E.A., van Eijck C.H., Bruno M.J., van der Harst E., Kubben F.J., Gerritsen J.J., Greve J.W., Gerhards M.F., de Hingh I.H. (2010). Preoperative biliary drainage for cancer of the head of the pancreas. N. Engl. J. Med..

[10] Nimptsch U., Krautz C., Weber G.F., Mansky T., Grutzmann R. (2016). Nationwide In-hospital Mortality Following Pancreatic Surgery in Germany is Higher than Anticipated. Ann. Surg..

[11] Clavien P.A., Barkun J., de Oliveira M.L., Vauthey J.N., Dindo D., Schulick R.D., de Santibañes E., Pekolj J., Slankamenac K., Bassi C. (2009). The Clavien-Dindo classification of surgical complications: Five-year experience. Ann. Surg..

[12] Ze X., Duncan S.H., Louis P., Flint H.J. (2012). *Ruminococcus bromii* is a keystone species for the degradation of resistant starch in the human colon. ISME J..

[13] Louis P., Flint H.J. (2017). Formation of propionate and butyrate by the human colonic microbiota. Environ. Microbiol..

[14] Sandini M., Honselmann K., Birnbaum D., Gavazzi F., Chirica M., Moutardier V., Keck T., Zerbi A., Gianotti L. (2018). Preoperative Biliary Stenting and Major Morbidity After Pancreatoduodenectomy: Does Elapsed Time Matter?: The FRAGERITA Study Group. Ann. Surg..

[15] Wahlstrom A., Sayin S.I., Marschall H.U., Backhed F. (2016). Intestinal Crosstalk between Bile Acids and Microbiota and Its Impact on Host Metabolism. Cell Metab..

[16] Islam K.S., Fukiya S., Hagio M., Fujii N., Ishizuka S., Ooka T., Ogura Y., Hayashi T., Yokota A. (2011). Bile acid is a host factor that regulates the composition of the cecal microbiota in rats. Gastroenterology.

[17] Ridlon J.M., Kang D.J., Hylemon P.B., Bajaj J.S. (2014). Bile acids and the gut microbiome. Curr. Opin. Gastroenterol..

[18] Heckler M., Mihaljevic A.L., Winter D., Zhou Z., Liu B., Tanaka M., Heger U., Michalski C.W., Büchler M.W., Hackert T. (2020). *Escherichia coli* Bacterobilia Is Associated with Severe Postoperative Pancreatic Fistula After Pancreaticoduodenectomy. J. Gastrointest. Surg..

[19] Yang Y., Duan Y., Su C., Sheng J., Zhu L., Xie Y., Liu H., Tang N., Qiu Y., Lu C. (2025). The impact of preoperative biliary drainage on bile colonization of patients undergoing pancreaticoduodenectomy. Ann. Med..

[20] Campbell C., McKenney P.T., Konstantinovsky D., Isaeva O.I., Schizas M., Verter J., Mai C., Jin W.-B., Guo C.-J., Violante S. (2020). Bacterial metabolism of bile acids promotes generation of peripheral regulatory T cells. Nature.

[21] Hang S., Paik D., Yao L., Kim E., Trinath J., Lu J., Ha S., Nelson B.N., Kelly S.P., Wu L. (2019). Bile acid metabolites control T(H)17 and T(reg) cell differentiation. Nature.

[22] Cai J., Sun L., Gonzalez F.J. (2022). Gut microbiota-derived bile acids in intestinal immunity, inflammation, and tumorigenesis. Cell Host Microbe.

[23] Jia W., Xie G., Jia W. (2018). Bile acid-microbiota crosstalk in gastrointestinal inflammation and carcinogenesis. Nat. Rev. Gastroenterol. Hepatol..

[24] Stein-Thoeringer C.K., Renz B.W., De Castilhos J., von Ehrlich-Treuenstätt V., Wirth U., Tschaidse T., Hofmann F.O., Koch D.T., Beirith I., Ormanns S. (2025). Microbiome Dysbiosis With Enterococcus Presence in the Upper Gastrointestinal Tract Is a Risk Factor for Mortality in Patients Undergoing Surgery for Pancreatic Cancer. Ann. Surg..

[25] Zaborin A., Smith D., Garfield K., Quensen J., Shakhsheer B., Kade M., Tirrell M., Tiedje J., Gilbert J.A., Zaborina O. (2014). Membership and behavior of ultra-low-diversity pathogen communities present in the gut of humans during prolonged critical illness. mBio.

[26] Harnoss J.C., Halm D., Weber S., Kinny-Köster B., Heckler M., Klotz R., Kalkum E., Harnoss J.M., Musa J., Probst P.M. (2025). A New Gold Standard? Impact of Broad-spectrum Penicillin-based Antibiotic Prophylaxis on Outcome After Pancreatoduodenectomy—Results of a Systematic Review and Meta-analysis (PROSPERO CRD42024559197). Ann. Surg..

[27] Garcia-Ochoa C., McArthur E., Skaro A., Leslie K., Hawel J. (2021). Pre-operative stenting and complications following pancreatoduodenectomy for pancreatic cancer: An analysis of the ACS-NSQIP registry. Surg. Endosc..

[28] Elmunzer B.J., Maranki J.L., Gómez V., Tavakkoli A., Sauer B.G., Limketkai B.N., Brennan E.A., Attridge E.M., Brigham T.J., Wang A.Y. (2023). ACG Clinical Guideline: Diagnosis and Management of Biliary Strictures. Am. J. Gastroenterol..

