# Supplementary material for: Tumor Location and Preoperative Biliary Stenting Shape Gut Microbiome Diversity in Pancreatic Cancer
Source: Cancers (Basel). 2026 Aug 14;18(16):2617. doi: 10.3390/cancers18162617 (PMC13511520; doi:10.3390/cancers18162617)
Supplement: Supplementary file 1 [file cancers-18-02617-s001.zip › cancers-4366906-supplementary.pdf]

Supplementary data, Table S1: Patients characteristics of the cohort with pancreatic head cancer.

PBS, preoperative biliary stent; BMI, body mass index in kg/m^2, ASA, Grading in American Society of Anesthesiologists; PPI, proton pump inhibitor

	Overall cohort (n=40)	Patients with PBS (n=14)	Patients without PBS (n=26)	p-value
**Age (median)**	64 (48-60)	64 (48-80)	63.5 (52-78)	0.9
**Gender (male)**	28 (70.0%)	16 (61.5%)	12 (85.7%)	0.1
**Weight (median)**	78.1 (48-105)	78 (48-102.6)	83.5 (64-105)	0.3
**BMI (median)**	25.4 (17.6-32.5)	25.5 (17.6-31.7)	25.4 (21.8-32.5)	0.7
**ASA**				0.6
1	1 (2.6%)	1 (4.2%)	0	
2	21 (55.3%)	12 (50.0%)	9 (64.3%)	
3	16 (42.1%)	11 (45.8%)	5 (35.7%)	
**Smoker**				0.3
no	37 (92.5%)	25 (96.2%)	12 (85.7%)	
yes	3 (7.5%)	1(3.8%)	2 (14.3%)	
**Alcohol**				0.6
no	39 (97.5%)	24 (92.3%)	12 (85.7%)	
yes	1 (2.5%)	2 (7.7%)	2 (14.3%)	
**Diabetes**				0.5
no	27 (67.5%)	19 (73.1%)	8 (57.1%)	
yes	13 (32.5%)	7 (26.9%)	6 (42.9%)	
**Metformin**				0.4
no	34 (85.0%)	23 (88.5%)	11 (78.6%)	
yes	6 (15.0%)	3 (11.5%)	3 (21.4%)	
**PPI**				0.4
no	26 (65.0%)	18 (69.2%)	8 (57.1%)	
yes	14 (35.0%)	8 (30.8%)	6 (42.9%)	

Supplementary data, Table S2: Surgical and clinical outcomes with pancreatic head cancer.

PBS, preoperative biliary stent; CA 19-9, carbohydrate antigen 19-9; CEA, carcinoembyonic antigen* According to Clavien-Dindo classification within 30 days after discharge

	Overall cohort	Patients with PBS (n=14)	Patients without PBS (n=26)	p-value
**CRP (preoperative; median)**	2.2 (1-54)	3 (1-54)	2.1 (1.9-12.8)	0.7
**Bilirubin (preoperative; median)**	0.7 (0.3-13.9)	0.9 (0.4-2.4)	0.6 (0.3-13.9)	0.7
**CA 19-9 (preoperative; median)**	59.9 (0.9-50511)	28.5 (0.9-50511)	78.9 (2.5-6484.2)	0.3
**CEA (preoperative; median)**	1.2 (0.2-57.9)	1 (0.4-57.9)	1.5 (0.2-9)	0.4
**Tumor stage**				0.6
I	12 (30.0%)	3 (21.4%)	9 (34.6%)	
II	10 (25.0%)	4 (28.6%)	6 (23.1%)	
III	10 (25.0%)	5 (35.7%)	5 (19.2%)	
IV	8 (20.0%)	2 (14.3%)	6 (23.1%)	
**Neoadjuvant chemotherapy**				0.6
No	33 (82.5%)	11 (78.6%)	22 (84.6%)	
Yes	7 (17.5%)	3 (21.4%)	4 (15.4%)	
**Time intervall between intervention and surgery (in days, median)**		29 (10-164)		
**Surgery**				0.4
Partial pancreaticoduodenectomy	28 (70.0%)	10 (71.4%)	18 (69.2%)	
Total pancreatectomy	6 (15.0%)	2 (14.3%)	4 (15.4%)	
exploration	6 (15.0%)	2 (14.3%)	4 (15.4%)	
**Duration of the surgical procedure (median, in min)**	378 (36-662)	375 (36-539)	382 (55-662)	0.8
**Blood loss (median, in ml)**	800 (10-6000)	800 (200-2600)	800 (10-6000)	0.8
**Intraoperative smear**				<0.001
Negative	10 (25.0%)	0	10 (38.5%)	
Positive	17 (42.5%)	12 (85.7%)	5 (19.2%)	
No smear	13 (32.5%)	2 (14.3%)	11 (42.3%)	
**Complications***				0.04
> 3a	5 (12.5%)	4 (28.6%)	1 (3.8%)	

Supplementary data, Table S3: Multivariable analysis to analyse independent factor for low alpha diversity.

Positive predictive factors was preoperative biliary stent.

CI, confidence interval; BMI, body mass indey; CA 19-9, carbohydrate antigen 19-9; CEA, carcinoembyonic antigen

	Odds Ratio	Lower.CI	Upper.CI	p-value
BMI	0.3	0.07	1.8	0.2
Diabetes	0.5	0.1	2.3	0.3
Bilirubin	3.0	0.6	15.5	0.2
CA 19-9	0.5	0.07	3.6	0.5
Intraoperatie smear	0.5	0.07	3.5	0.5
Preoperative biliary stent	8.9	1.1	74.0	0.04
